# Integration matters: Combining socio-cultural and biophysical methods for mapping ecosystem service bundles

**DOI:** 10.1007/s13280-023-01830-7

**Published:** 2023-02-13

**Authors:** Jarrod Cusens, Alicia D. Barraclough, Inger Elisabeth Måren

**Affiliations:** 1 Department of Biological Sciences, University of Bergen, Thormøhlens Gate 53A, 5006 Bergen, Norway; 2 Centre for Sustainable Area Management (CeSAM), University of Bergen, Bergen, Norway; 3 UNESCO Chair on Sustainable Heritage and Environmental Management, University of Bergen, Bergen, Norway

**Keywords:** Biocultural diversity, Biosphere Reserve zonation, Ecosystem service bundles, Socio-cultural values, UNESCO Biosphere Reserves

## Abstract

Ecosystem services (ESs) play an important role in sustainable landscape management. People value ESs in diverse ways encompassing social and ecological domains and we need to bring these different values together. We used social-cultural and biophysical methods to map a diverse set of ESs at two spatial scales in a UNESCO Biosphere Reserve in Norway. The ESs bundled into three distinct social–ecological system archetypes which were similar in their distribution and relative ES values at both spatial scales. The bundles were also well matched to relative ESs values of the Biosphere Reserve zones (core, buffer, and transition) indicating that the bundles capture the social–ecological systems of the zones. We argue that it is important to consider the social–ecological context of the zones to provide sufficient knowledge to inform management. Our work has the capacity to contribute to sustainable land management that takes biocultural values into consideration.

**Supplementary Information:**

The online version contains supplementary material available at 10.1007/s13280-023-01830-7.

## Introduction

Humans are intricately linked with, and are entirely reliant on nature and the ecosystem services (ES) that we co-produce with nature including clean water, fresh air and food, and intangible benefits like mental well-being (e.g. Millennium Ecosystem Assessment 2005; Bratman et al. 2012). This reliance is clearly reflected in the widespread mark we have left on the planet, with 69–76% of Earth’s surface showing evidence of human modification, much of which is the result of our co-production of ES with nature (Ellis and Ramankutty 2008). The ES concept is now mainstream in social–ecological research and increasingly used in policies and land-use planning decisions from local to continental scales (Maes et al. 2012; Schröter et al. 2016; Schubert et al. 2018; Longato et al. 2021). In the last decade there have been significant conceptual shifts in ES thinking driven in part by the work of the Intergovernmental Science-Policy Platform on Biodiversity and Ecosystem Services and others (e.g. Mace 2014; Martín-López et al. 2014; Díaz et al. 2015; IPBES 2019). These shifts bring about a wholistic view of ES by acknowledging the plurality of contributions that nature makes to our wellbeing and recognising that our values for nature are not only instrumental, but are also intrinsic and relational (Díaz et al. 2015; Pascual et al. 2017; Kenter 2018; Maes et al. 2018; Kadykalo et al. 2019). Indeed, Nature’s Contributions to People (NCP) is a term introduced by IPBES to capture those multiple values of nature from a broader range of society (Díaz et al. 2015, 2018). Although there has been substantial debate about how ES and NCP differ, it is overall reasonable to acknowledge that they are broadly similar, particularly in recent ES research (see Kadykalo et al. 2019). We therefore use the term ES throughout but recognise that some differences between the terms exist.

### Multiple values of landscapes

Landscapes develop through interactions between nature and people through cultural, social, and economic practices (Olwig 2007). Focussing on either biophysical or social–cultural values in sustainability problems will fail to capture the full breadth of values offered by landscapes (Meyfroidt et al. 2022). Integrating different value types and ways of measuring (e.g. biophysical and socio-cultural) into ES assessments is an important step to implementing contemporary ES thinking into governance, management, and planning. Assessment and mapping of ES studies have often been constrained to either biophysical or economic approaches, although there are an increasing number of studies using socio-cultural and pluralistic methods (Martín-López et al. 2019; Schutter and Hicks 2021). Biophysical approaches have contributed substantially to the understanding of the spatial distributions and interactions between ES, particularly provisioning, and regulating and maintenance ES (Chan and Satterfield 2020). These biophysical methods link biological and physical attributes of the landscape to ES supply with varying degrees of complexity from simple proxy-based approaches assigning ES values to land use–land cover (LULC) types, to more complex process-based models that incorporate a diversity of parameters such as geochemistry, climate and biotic characteristics like plant traits (reviewed by Lavorel et al. 2017). However, biophysical methods have been somewhat limited in their capacity to map cultural ES and are lacking in their ability to capture social–cultural values of ES (Brown and Fagerholm 2015; Chan and Satterfield 2020).

We adopt the definition of socio-cultural values formulated by Scholte et al. (2015, p. 68) as “the importance people, as individuals or as a group, assign to (bundles of) ESs”. Methods that elicit the values that people assign to ES are therefore considered socio-cultural in our interpretation. Amongst studies using socio-cultural methods for ES mapping, Public Participation GIS (PPGIS) has become prominent in the literature (e.g. Plieninger et al. 2013; Brown and Fagerholm 2015; Fagerholm et al. 2019). The potential of PPGIS has been highlighted to address deficits in other mapping methods for cultural ES (Crossman et al. 2013; Brown and Fagerholm 2015) and several studies have combined PPGIS for cultural ES with other methods for provisioning and/or regulating and maintenance ES (Bagstad et al. 2017; Lin et al. 2017; Rolo et al. 2021; Zhao et al. 2021). These studies provide a basis for progressing research into relationships between multiple ES across all ES categories within landscapes for planning and management applications.

### Ecosystem service bundles

Landscapes provide different ES, or sets of ES, depending on their configuration such as the areal extent of the ecosystems, the geological landforms, and type and intensity of human intervention within them (Bennett et al. 2009). ES bundles—“sets of ecosystem services that repeatedly appear together across space or time” (Raudsepp-Hearne et al. 2010, p. 5242) are widely used to assess the multifunctionality of landscapes and/or ecosystems (e.g. Raudsepp-Hearne et al. 2010; Turner et al. 2014; Queiroz et al. 2015), although it has been pointed out that bundles are not synonymous with multifunctionality (Saidi and Spray 2018). In a review Meacham et al. (2022) identified five benefits of using ES bundle analyses related to (1) simplifying analysis, (2) simplifying management, (3) developing practical social–ecological theory, (4) filling data gaps, and (5) acting as a bridging tool. In addition, ES bundles can assist in identifying social–ecological system archetypes within a landscape (Hamann et al. 2015). Since ES are co-produced by people and nature (Spangenberg et al. 2014), ES bundles can be recognised as distinct social–ecological systems that have emerged though complex interactions and feedbacks between social and ecological systems (Folke et al. 2010; Reyers et al. 2013; Hamann et al. 2015). These social–ecological system archetypes can provide important information to guide conservation planning and management, particularly in light of modern framing of conservation as ‘People and Nature’ (cf. Mace 2014).

### Ecosystem services across scales

From a planning and management perspective it makes sense to map ES values and subsequent ES bundles at the spatial scale at which management decisions are made, and many studies have taken this approach and mapped ES bundles at the municipality scale (e.g. Raudsepp-Hearne et al. 2010; Queiroz et al. 2015; Malmborg et al. 2021). However, although governance decisions are often made at larger scales many ES are effectively produced and managed at much smaller scales such as the farm or field level. Therefore, mapping ES at a single scale may lead to a spatial mismatch between the scale at which ES are mapped and bundled, and the scale at which they are produced, managed, and/or governed (Raudsepp-Hearne and Peterson 2016). This scale-mismatch means that management actions to enhance a particular ES at one scale can result in trade-offs with other ES at different scales (Raudsepp-Hearne and Peterson 2016). Mapping and identifying ES bundles at multiple scales to account for the different scales that ES are produced, managed, and/or governed can contribute to addressing issues that may arise with such mismatches (Scholes et al. 2013).

### Ecosystem services in UNESCO Biosphere Reserves

**Fig. 1:**
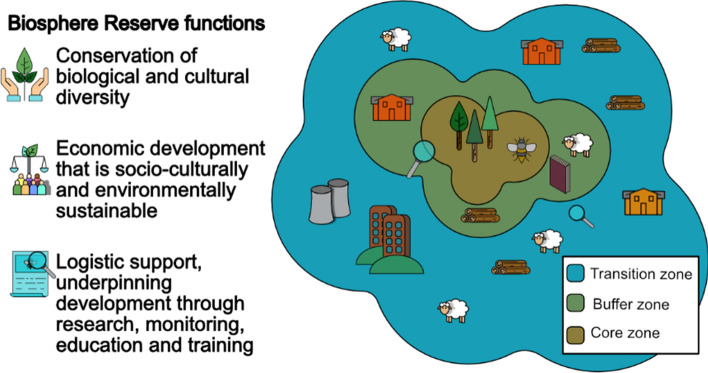
Conceptual representation of the UNESCO Biosphere Reserve zonation and the three functions of Biosphere Reserves. Adapted from https://en.unesco.org/biosphere/

The United Nations Educational, Scientific and Cultural Organization (UNESCO) Biosphere Reserves (BRs) provide succinct case studies for exploring ES assessment, governance, and management in social–ecological landscapes. Biosphere Reserves are explicitly recognised as both sources and stewards of ES (UNESCO 2017) emphasised by recent requirements to report on the state of ES in periodic reviews. ES assessments can therefore be an important tool for monitoring success of BR objectives (Vasseur and Siron 2019; Palliwoda et al. 2021). Secondly, BRs are divided into three distinct zones; core, buffer, and transition/development (Fig. 1). Zonation provides the basis for achieving the three primary BR functions of (i) biocultural diversity conservation, (ii) sustainable development, and (iii) logistic support for research, monitoring, education and training (Fig. 1), and thus we can expect that zones provide different ES (Palliwoda et al. 2021). We use biocultural diversity as defined by Maffi (2005, p. 602) as the “diversity of life in all its manifestations—biological, cultural, and linguistic—which are interrelated within a complex socio-ecological adaptive system”.

Several studies have mapped the spatial distribution of ES values in BRs using both biophysical methods (e.g. Kermagoret and Dupras 2018; Poikolainen et al. 2019) and socio-cultural methods (e.g. Plieninger et al. 2013; Cusens et al. 2022). However, few studies have the BR zonation explicitly in their analyses (but see Castillo-Eguskitza et al. 2019; Palliwoda et al. 2021; Cusens et al. 2022). Palliwoda et al. (2021) explicitly mapped and analysed the differences in ES co-production across the zones of 137 European BRs finding that ES co-production does not always match with the objectives of zonation within BRs. Castillo-Eguskitza et al. (2019) mapped biophysical and monetary ES values in Urdaibai BR, Spain, and assessed the coincidence between the two value types within the BR zones. Although these two studies highlight the value of zone-specific ES valuation for assessing BR goals and objectives, both consider zones as an aggregate of each zone type (i.e. core, buffer, and transition) within a BR. However, many BRs do not comprise a single core or buffer zone which means that aggregate ES values across all core or buffer zones may fail to capture the idiosyncrasies in ES values across each zone type. A recent study used PPGIS to map social-cultural values of the zones in Nordhordland Biosphere Reserve in Norway and found that values within zone types were quite variable pointing to the need for multiscale assessment of ES in BR zones (Cusens et al. 2022).

In our case study we combine biophysical and social-cultural methods to map 14 ES within Nordhordland Biosphere Reserve (NBR), a recently designated BR in Norway (Kaland et al. 2018). We first ask how ES provision varies across the BR zones in NBR. Second, we ask if there are distinct ES bundles within NBR, and if the spatial scale of bundles (municipal and grid) influences the relative ES values and spatial distribution of the bundles. Third, we ask how the ES bundles are captured within the BR zones in relation to their distribution and relative ES values. Finally, we discuss the potential applicability of ES bundles that integrate biophysical and socio-cultural methods to inform planning and management of biocultural diversity conservation in BRs, and other social–ecological systems more broadly.

## Materials and methods

### Study area

**Fig. 2:**
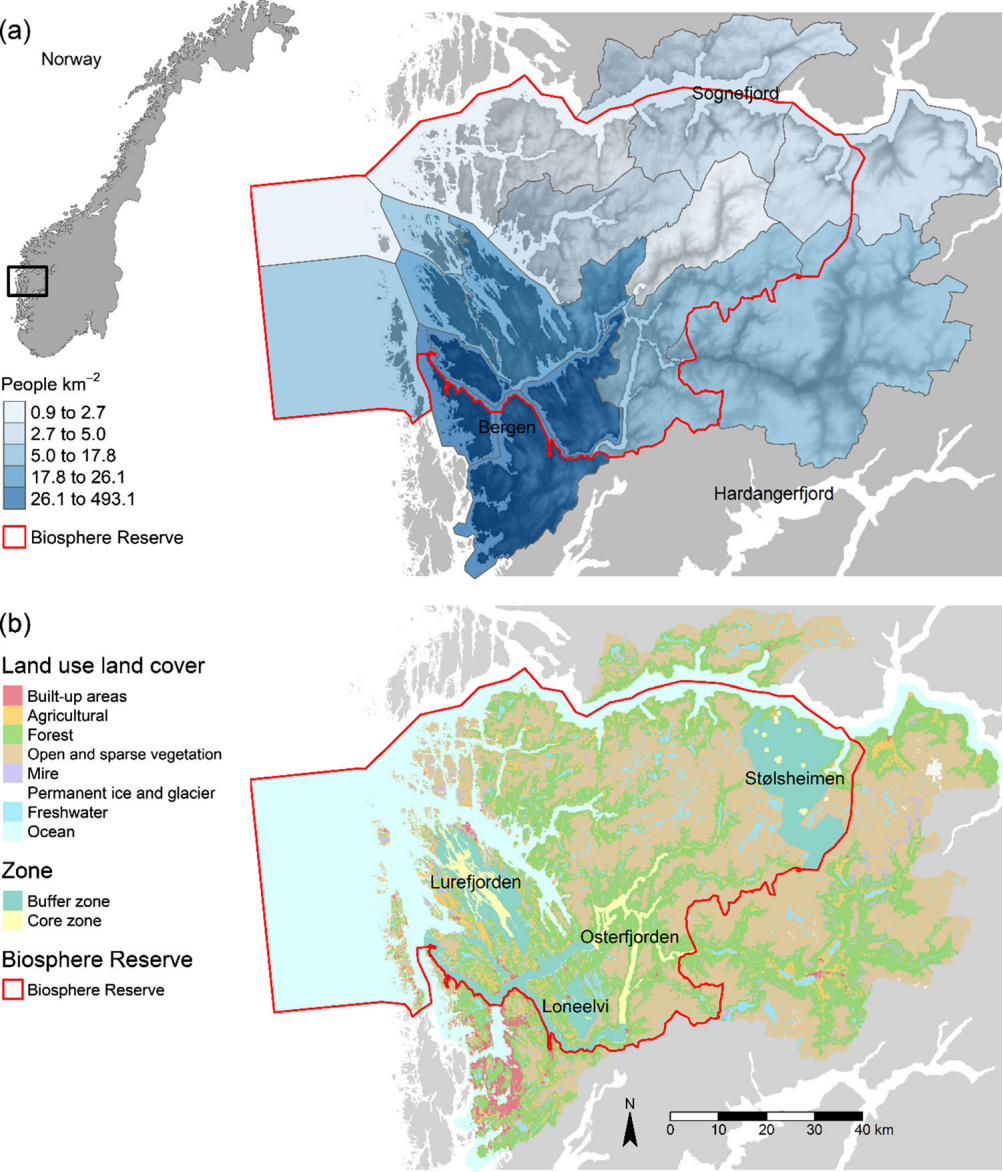
**a** Location and population densities of the municipalities, and **b** land use–land cover and the location of the different zones in Nordhordland UNESCO Biosphere Reserve at the west coast of Norway

Nordhordland UNESCO Biosphere Reserve (hereafter NBR) is located on the west coast of Norway covering c. 6700 km^2^ stretching from the open Atlantic Ocean in the west, through the low-lying coastal flats on the west coast, up to the mountains in the east (Fig. 2a). The terrestrial landcover comprises predominantly ‘open and sparse vegetation’ (34%) and forest (24%; Fig. 2b) with agricultural land making up 3%. Marine environments are cover a large spatial extent (29%) including open ocean and extensive fjord systems. The region is characterised by a mild wet-temperate oceanic climate with high mean annual rainfall (2400 mm/year). There is a strong west–east precipitation gradient from coast to the mountains with the coastal areas receiving 1300 mm/year whilst the upland areas receive 3000 mm/year. Mean temperature of the warmest and coldest months is 13.0–14.5 °C and 3.0–3.0 °C, respectively in the coastal areas. The administrative units comprise nine municipalities that are contained entirely with the boundaries of NBR, as well as a further five that are partially within the boundaries (Fig. 2a). The permanent human population of the nine main municipalities is c. 54 000 concentrated in low-lying southwestern coastal areas in the settlements of Knarvik, Frekhaug, Valestrandfossen, Lindås, and Manger (Fig. 2a).

The zonation of NBR comprises four localities with a core and buffer zone associated with each (Fig. 2b; Kaland et al. 2018). The zones represent the major land- and seascapes in NBR including the coast and outer archipelago (Lurefjorden), the marine and terrestrial components on the outer fjords (Osterfjorden and Loneelvi River), and the inland mountain landscape (Stølsheimen; Fig. 2b). Each zonation locality has its own unique characteristics encompassing various components of the biocultural diversity found in NBR including cultural heritage monuments and upland summer farms at Stølsheimen, cultural landscapes in the buffer zones of Loneelvi and Lurefjorden, and important biodiversity and research sites in the core areas of Lurefjorden and the National Salmon Fjord in Osterfjorden.

### Ecosystem services typology

The ES typology was developed in three steps. First, we used the NBR UNESCO application document (Kaland et al. 2018) to identify locally relevant ES. Second, we referred to published literature on ES mapping to find ES not previously identified. Finally, we used a workshop with local stakeholders to test the typology and identify any ES we had missed. We attempted to link our typology to the Common International Classification of Ecosystem Services version 5.1 (CICES; Haines-Young and Potschin 2018) and IPBES NCPs (Díaz et al. 2018) wherever possible. However, there are some cultural ES in our typology not strictly linked to single classes within CICES (e.g. inspiration, spiritual, and aesthetic) because the statements we used in PPGIS survey (see below) needed to be locally relevant and understandable to non-experts (Cusens et al. 2022). In addition, water yield has no equivalent within IPBES NCPs. The final typology contained 14 ES comprising five regulating and maintenance, four provisioning, and six cultural ES (Table 1).

**Table 1:** An overview over the 14 ecosystems services (ES), the service providing areas (SPAs), and the methods used for mapping them

ES	SPAs	Method/index	Units	References
Cultural				
Appreciation of biodiversity	All areas	PPGIS and MaxEnt modelling	Unitless (0–1)	Sherrouse et al. (2011)
Cultural heritage	All areas	PPGIS and MaxEnt modelling	Unitless (0–1)	Sherrouse et al. (2011)
Hunting and fishing^a^	All areas	PPGIS and MaxEnt modelling	Unitless (0–1)	Sherrouse et al. (2011)
Inspiration, spiritual and aesthetic	All areas	PPGIS and MaxEnt modelling	Unitless (0–1)	Sherrouse et al. (2011)
Outdoor recreation	All areas	PPGIS and MaxEnt modelling	Unitless (0–1)	Sherrouse et al. (2011)
Wild plant, berries and mushrooms^a^	All terrestrial areas	PPGIS and MaxEnt modelling	Unitless (0–1)	Sherrouse et al. (2011)
Regulating and maintenance				
Avalanche protection	Forested areas	Avalanche Protection Index	Unitless (0–1)	Cordonnier, et al. (2014)
Global climate regulation	All areas	Sum of carbon stored in vegetation and soil	ton/ha	For example, Mitchell et al. (2021)
Habitat quality	All areas	Phenomenological model of LULC, landscape metrics and threats	Unitless (0–1)	For example, Ruas et al. (2021)
Soil retention capacity	Vegetated terrestrial areas	Revised Universal Soil Loss Equation	ton/ha/year	Quintas-Soriano et al. (2019) and Renard et al. (1991)
Water retention	All terrestrial areas	Water Retention Index	Unitless (1–10)	Vandecasteele et al. (2018)
Provisioning				
Animal fodder	All terrestrial areas	Statistical downscaling based on land cover	ton/ha/year	Crouzat et al. (2015) and Statistics Norway (2019)
Drinking water	Cultivated areas	InVEST water yield model	mm/ha/year	Sharp et al. (2020)
Timber and firewood	Forested areas	Species and site quality specific annual timber increment	m3/ha/year	Schröter et al. (2014)

^a^These two ES are classified as provisioning ES by CICES. However, we have classified them as cultural services, consistent with the socio-economic background of our study region (Reyes-García et al. 2015)

### Cultural ecosystem services

We used a web based PPGIS to collect socio-cultural values for ES in NBR in which participants mapped points related six cultural ES based on statements adapted from published PPGIS-ES studies (e.g. Fagerholm et al. 2016; Plieninger et al. 2019) capturing both use and subjective perceptions of socio-cultural values of ES (Scholte et al. 2015). For more information regarding the PPGIS survey please see Cusens et al. (2022). To model the distributions of cultural ES we use an approach similar to Sherrouse et al. (2014) using maximum entropy (MaxEnt) modelling with 10 spatially explicit social–ecological landscape characteristics at a 250 m resolution (distance from roads, buildings, and hiking trails, percentage cover of agricultural land, water, forest and open LULC types, and elevation, slope, and richness of LULC). The variables were identified from previous studies as well as additional variables considered important in NBR (Table S1; Sherrouse et al. 2014; Bagstad et al. 2017; Muñoz et al. 2020). For more detail on the modelling methods please refer to Appendix S1.

### Regulating and maintenance, and provisioning ecosystem services

We used several approaches to map regulating and maintenance, and provisioning ES including: (1) national statistics available at the municipality and/or regional level downscaled to a grid (e.g. fodder production); (2) LULC proxy-based models (e.g. carbon storage); and (3) process-based models (e.g. water regulation) (Table 1). Values of each ES were normalised to unitless values between zero and one to enable comparison amongst different ES. See Appendix S1 for more detail on methods for each ES and data sources used.

### Ecosystem services and Biosphere Reserve zonation

Similarly to Palliwoda et al. (2021), we assessed the levels of provision of ES in the BR zones by calculating the median values for each ES in each zone. Before extracting these values, we excluded all non-service providing areas for services provided by single ecosystem types (Table 1). For example, non-forested or cultivated land for timber and avalanche protection, and fodder, respectively. We plotted the relative ES median values amongst the three main zones and for each individual zone. To test for differences in ES supply we used pairwise Wilcoxon rank sum tests to test for differences of ES provision within each zone for the three main zones (i.e. core, buffer, transition) as well as only zones within the terrestrial or the marine environment. We made the pairwise comparisons between core vs. buffer, buffer vs. transition and core vs. transition for each ES.

### Ecosystem service bundles

We produced ES bundles at two spatial scales (1) using municipalities (mean = 422.6 km^2^) and (2) 250 × 250 m grid cells as the spatial units. For the municipality scale we aggregated the grid scale data and calculated the mean value for each ES per municipality. We excluded the municipalities with less than 30% of their area within NBR resulting in 10 entire and three partial municipalities for the bundle analysis. For the grid scale we used the values per grid cell. Bundles were produced following a similar methodology of many other studies (e.g. Raudsepp-Hearne et al. 2010; Saidi & Spray 2018; Quintas-Soriano et al. 2019; Malmborg et al. 2021). At both scales we first reduced the dimensionality of the dataset with principal component analysis and selected the number of components that explained at least 65% of the variance and applied varimax rotation. Finally, we used *k*-means clustering to assign either municipalities or grid cells to clusters. We then chose the best number of clusters using the ‘Elbow method’ on the varimax rotated factor loadings. After we had assigned municipalities or grid cells to clusters, we calculated the mean value for each ES per cluster and represented these using flower-petal diagrams. In addition to generating the bundles, we calculated the percentage cover of LULC types within each bundle at both scales to qualitatively describe the social–ecological characteristics of the bundles. Land cover alone has previously been shown to be a strong predictor of ES bundle distribution (Meacham et al. 2016; Rolo et al. 2021). In addition, to compare how the bundles overlap with the different BR zones, we calculated the spatial overlap between the zones and the bundles and report this as a proportion.

### Software

We used *R* (R Core Team 2021) for all data manipulation, analysis, and visualisation (Table 2).

**Table 2:** R packages (R Core Team 2021) used for data manipulation, analysis, and visualisation

Package	Analysis/task	References
*EMNeval*	Maximum entropy modelling	Kass et al. (2021) and Muscarella et al. (2014)
*factoextra*	Cluster analysis	Kassambara and Mundt (2020)
*ggplot*	Plotting	Wickham (2016)
*ggpubr*	Plotting and analysis	Kassambara (2020)
*landscapemetrics*	Landscape metrics calculation	Hesselbarth et al. (2019)
*psych*	Principal component analysis	Revelle (2021)
*raster*	Raster data	Hijmans (2020)
*sf*	Vector data	Pebesma (2018)
*spatialEco*	Kernel density calculation	Evans (2020)
*stars*	Raster data	Pebesma (2022)
*tidyverse*	General tidy workflow	Wickham et al. (2019)
*tmap*	Spatial plotting	Tennekes (2018)

## Results

### Ecosystem service distributions

**Fig. 3:**
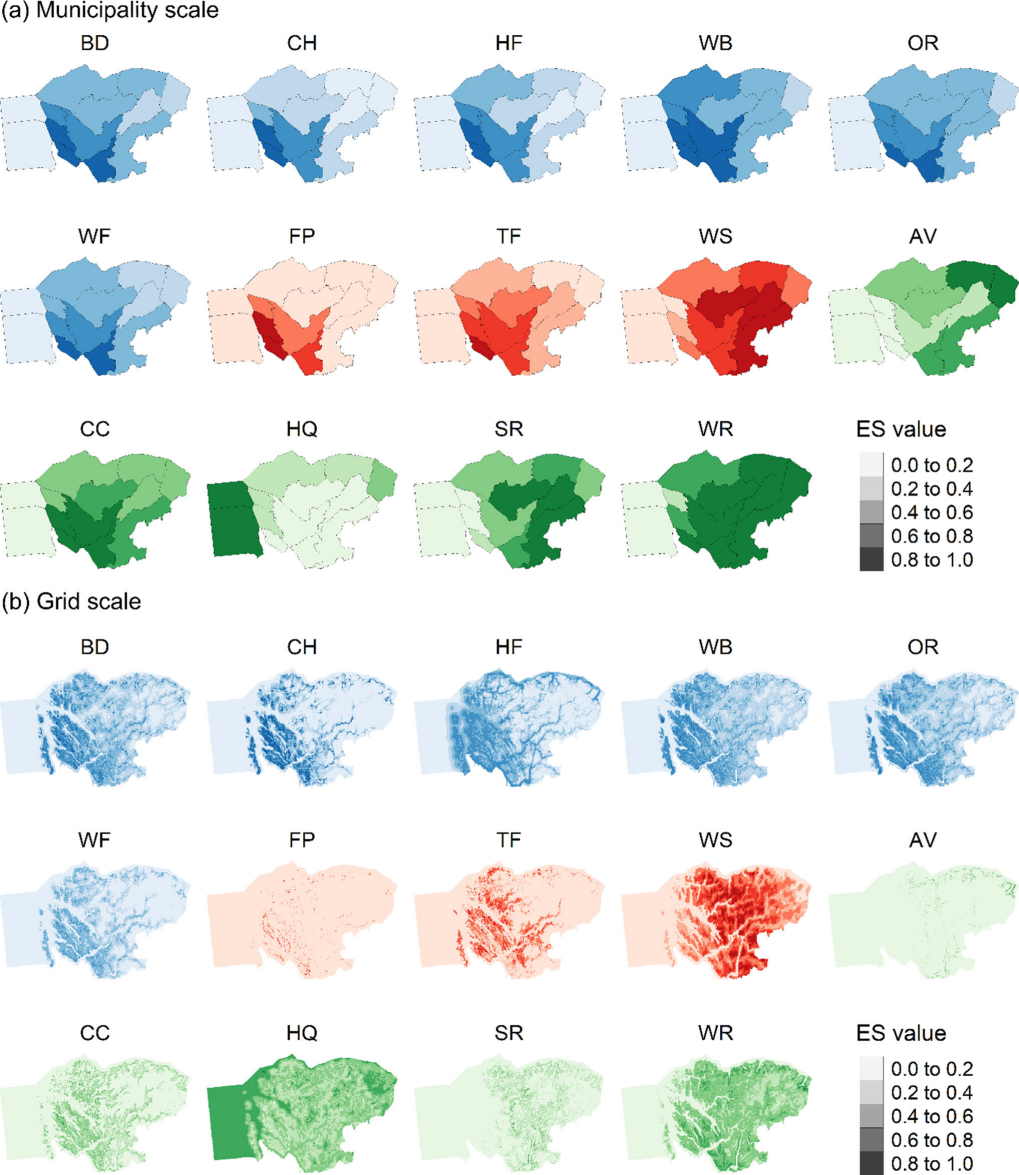
Distribution of normalised ecosystem service (ES) values of 14 ES at the **a** municipality scale and **b** the grid (250 × 250 m) scale in Nordhordland Biosphere Reserve. Cultural ES in blue, provisioning ES in red and, maintenance and resulting ES in green. *BD* appreciation of biodiversity, *CH* cultural heritage, *HF* hunting and fishing, *WB* inspiration, spiritual, and aesthetic, *OR* outdoor recreation, *WF* wild plants, berries or mushrooms, *FP* fodder production, *TF* timber production, *WS* water yield, *AV* avalanche protection, *CC* climate change mitigation, *HQ* habitat quality, *SR* sediment retention, *WR* water retention

In general, cultural, and provisioning ES tended to have higher values in the lowland coastal municipalities and terrestrial areas to the west although, water yield was highest in the eastern highland areas (Fig. 3). Regulating and maintenance ES were more spatially variable with water retention, avalanche protection, and sediment retention generally highest in the eastern upland areas, whereas habitat quality was highest in marine environments and climate change mitigation highest is the lowland terrestrial areas and municipalities. The grid scale mapping reveals some nuanced spatial variation not evident at the municipal scale including the very limited distributions of fodder production, avalanche protection, and sediment retention (Fig. 3b). Cultural heritage has highest values in the lowland areas within agricultural landscapes (Fig. 3b). In addition, the grid scale demonstrates the predominantly marine distribution of hunting and fishing indicating that this ES comprises predominantly fishing within NBR (Fig. 3b).

### Ecosystem services and Biosphere Reserve zonation

**Fig. 4:**
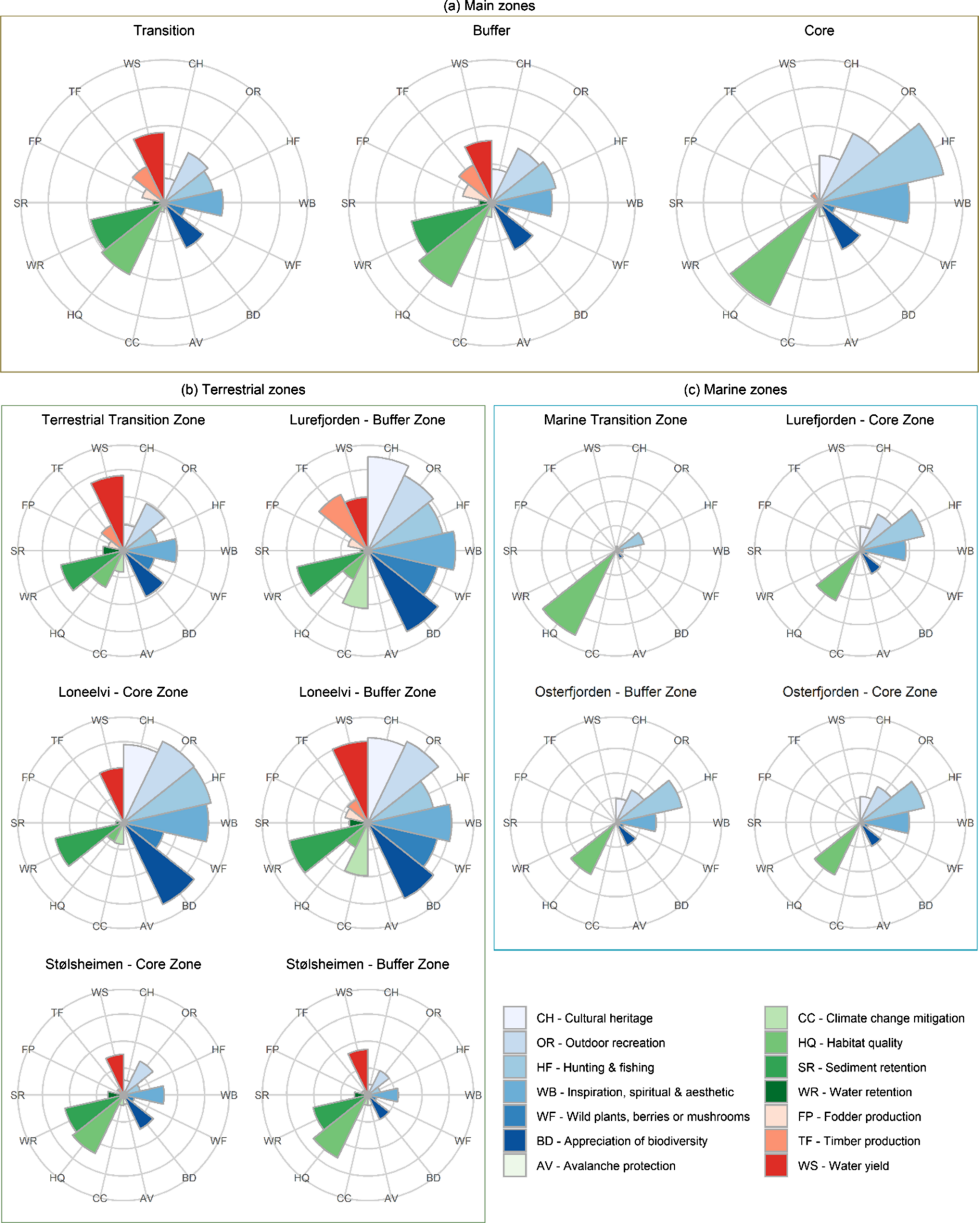
Median values of 14 ecosystem services in the **a** three main biosphere reserve zones, and individual zones separated into **b** terrestrial (and one freshwater) and **c** marine areas

Ecosystems service values were variable across the three main aggregate BR zones (i.e. core, buffer, transition; Fig. 4a). The distribution of ES values was similar in the buffer and transition zones whilst the core zone was quite distinct (Fig. 4a). Cultural ES tended to have higher values in the core zone and lowest values in the transition zones aside from wild plants, berries and mushrooms which was comparatively low in all zones. Provisioning ES values were lowest in the core zone and moderately higher in both buffer and transition zones. Habitat quality was consistently high in all three zones although highest in the core zone.

Amongst the individual zones paired core and buffer zones tended to have similar relative ES supply values (Fig. 4a and c). Specifically, the core and buffer zones within Loneelvi, Stølsheimen, and Osterfjorden core and buffer zones were similar. Further, there was a considerable contrast between terrestrial and marine zones overall (Fig. 4b and c). Provisioning and regulating and maintenance ES supply values were low in marine zones in comparison to the terrestrial zones. Marine zones were like each other although the marine transition zone had lower values for cultural ES than the marine core and buffer zones (Fig. 4c). Further, the ES supply values of aggregated core zones (Fig. 4a) were similar to the individual marine zones (Fig. 4c).

### Ecosystem service bundles

**Fig. 5:**
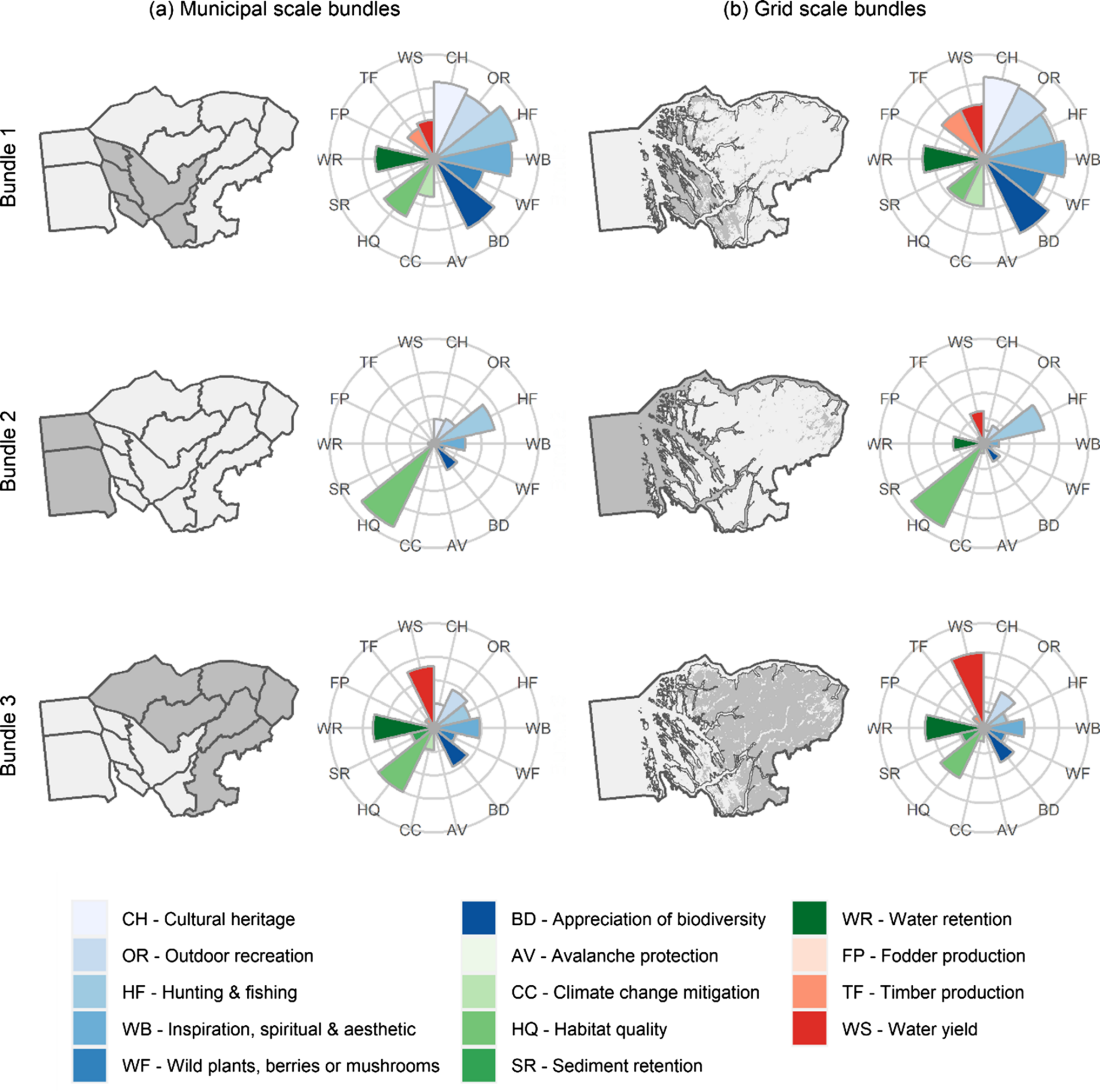
Distributions and mean values of 14 ecosystem services in the three bundles identified at **a** municipality and **b** grid (250 × 250 m) scales in Nordhordland Biosphere Reserve

We identified three ES bundles at both grid and municipal scales (Fig. 5). The spatial distribution of the bundles was similar with both scales consisting of a south-central located bundle (Bundle 1) in the higher populated areas and municipalities, a second (Bundle 2) to the west encompassing marine dominated areas and municipalities, and a third north-west located bundle (Bundle 3) in the more mountainous areas and municipalities (Fig. 5). The total area covered by the bundles differs at the two scales despite their similar spatial distributions (Table 3). The relative values of different ES of the bundles were very similar at both scales. Bundle 1 had high values for all cultural ES and moderate values for provisioning and, regulating and maintenance ES. Bundle 2 had high values for habitat quality and hunting and fishing. Bundle 3 had high values for water supply and moderate values for water retention and habitat quality (Fig. 5).

**Table 3:** The number of spatial units (municipalities or grid cells) and spatial area of the three ecosystems service bundles identified in Nordhordland UNESCO Biosphere Reserve

Bundle	No. spatial units	Area of bundle (km^2^)	Percent of bundle (%)
Municipality scale			
1	5	1459.85	22.1
2	2	1462.20	22.1
3	6	3688.03	55.8
Grid scale			
1	22 225	1389.10	20.5
2	39 833	2489.60	36.8
3	46 132	2883.30	42.6

### Comparing zones and bundles

**Fig. 6:**
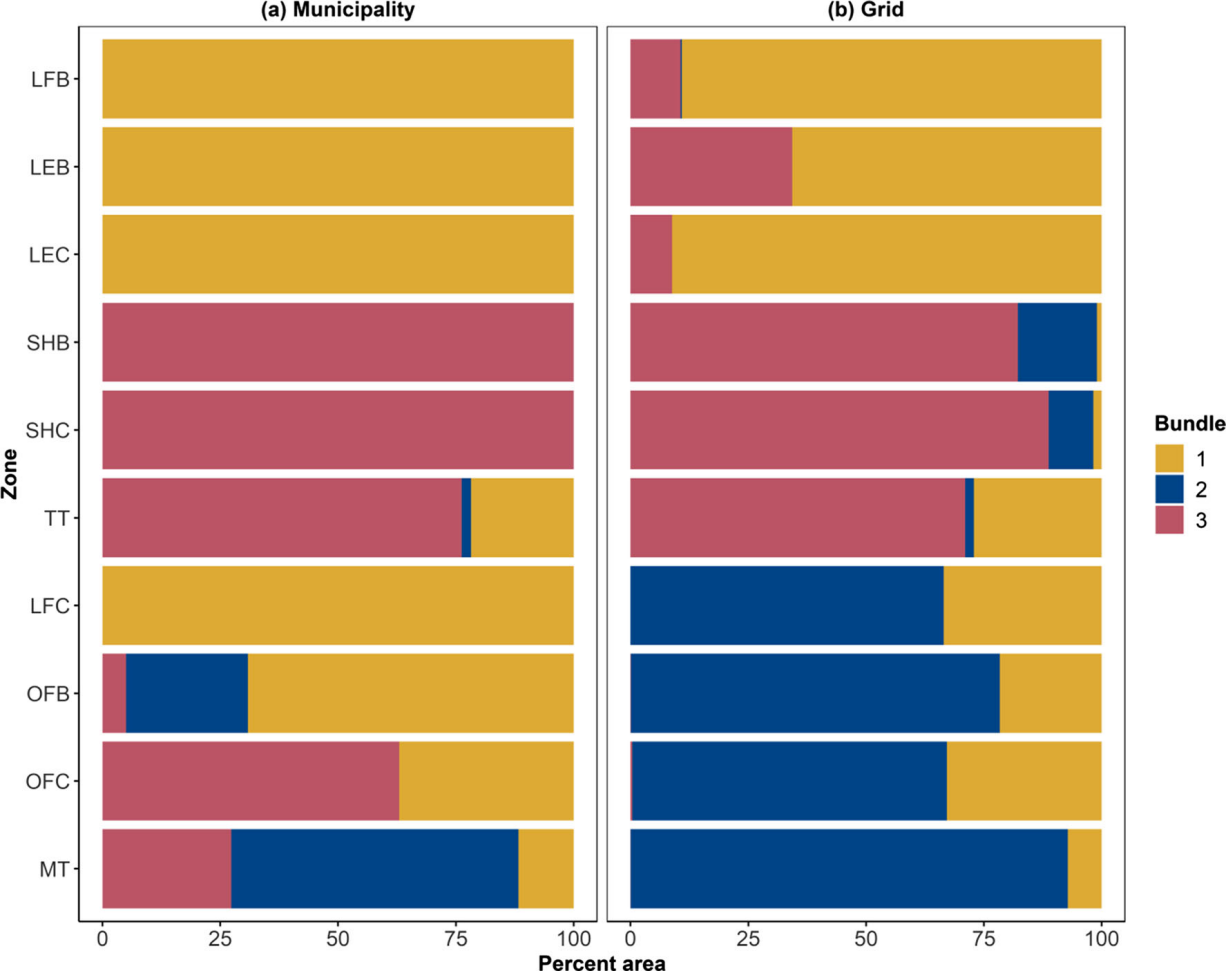
Proportional bundle composition of each zone in Nordhordland UNESCO Biosphere Reserve at the **a** municipality and **b** grid scales. *MT* marine transition, *OFC* Osterfjorden core, *OFB* Osterfjorden buffer, *LFC* Lurefjorden core, *TT* terrestrial transition, *SHC* Stølsheimen core, *SHB* Stølsheimen buffer, *LEC* Loneelvi core, *LEB* Loneelvi buffer, *LFB* Lurefjorden buffer

The relative ES values in Bundle 1 was most like buffer zone of Lurefjorden, and both the core and buffer zones of Loneelvi, Bundle 2 was most similar to the marine transition zone and to a lesser extent the core and buffer zones of the other marine dominated areas, and Bundle 3 was most similar to the terrestrial transition zone and to a lesser extent the core and buffer zones of Stølsheimen (Figs. 4, 5). An overlay of the areal extent of the bundles and the zones revealed that the lowland terrestrial and freshwater zones comprise entirely or almost entirely of Bundle 1 at the municipal and grid scales respectively (Fig. 6). Similarly, the terrestrial transition and upland core and buffer zones comprise predominantly Bundle 3 at both scales (Fig. 6). At the grid scale all marine zones comprise predominantly Bundle 2 (Fig. 6). In marine zones at the municipal scale, however, there is substantial variation in the bundle composition of the zones (Fig. 6). Lurefjorden core and Osterfjorden buffer comprise predominantly Bundle 2, whereas Osterfjorden core is predominantly within Bundle 3.

## Discussion

### Integrated mapping matters

We combined socio-cultural and biophysical methods to map 14 ES in a UNESCO Biosphere Reserve. The mapped ES were then used to compare ES supply across zones and to assess bundles of ES within the BR. Integrating socio-cultural and biophysical methods revealed some important insights about the distribution of ES values amongst the zones and the bundles we identified. The socio-cultural method for mapping cultural ES adds an important dimension to the mapping, and many ES would be unrepresented, and the composition of ES bundles would be substantially different if only biophysical methods were used (Bagstad et al. 2016). This is emphasised in our finding of a predominance and high diversity cultural ES in zones and bundles in areas close to more human modified landscapes (see for example Bundle 1 vs. Bundle 3 in Fig. 3). Biophysical methods alone limit the number and types of cultural ES that could be assessed due to limited knowledge on their distributions in different contexts. However, if only socio-cultural methods were used, we would fail to capture the distribution and values of a diverse set of ES beyond cultural ES alone. Firstly, there would be limited information on regulating and maintenance ES, since values for this ES class are typically mapped at low proportions relative to other ES in PPGIS studies, especially when compared to cultural ES (e.g. Garcia-Martin et al. 2017; Fagerholm et al. 2019; Cusens et al. 2022). Secondly, places further from human settlements would be underrepresented in our ES maps because low populated areas in the mountains had very few places mapped in our PPGIS study (41 or 3155 places) comprising almost entirely outdoor recreation (Cusens et al. 2022). This stems from both spatial discounting, where people map more places close to home (e.g. Brown and Kyttä, 2014; Fagerholm et al. 2019) and that people tend to not perceive complex processes involved in regulating and maintenance ES (Scholte et al. 2015). Our approach contributes to a growing literature and calls to bring together multiple approaches to ES assessment and mapping (e.g. Martín-López et al. 2019; Chan and Satterfield 2020). We show how mixed-methods can help highlight places with high cultural ES values as well as provisioning and maintenance and supporting ES values, providing a more holistic approach to ES mapping.

### The spatial scale of the social–ecological system archetypes

Each of the three bundles we identified in NBR were distinct in their relative ES values. At the same time, bundles at different spatial scales were remarkably similar in both relative ES values and in their distribution. The consistency of the bundles across scales is the result of strong and clear social–ecological gradients characterised by both the land- and water-forms, land-use intensity, and the human populations and associated infrastructure. We interpret the bundles in our study as three distinct social–ecological systems archetypes comprising the low-lying ‘coastal flats’ with higher population density and mixed LULC types (Bundle 1), of predominantly marine and fjord dominated systems (Bundle 2), and the less populated mountainous regions in the east comprising predominantly open vegetation and to a lesser extent forest (Bundle 3) (see Appendix S3, Fig. S4 for proportions of LULC types in each bundle). In regard to scale, our results contrast with Raudsepp-Hearne and Peterson (2016) who found clearer differences in ES values between their smallest grid-scale (1 km^2^) and larger municipality scales. The spatial extent in their study was significantly smaller than ours (c. 700 vs. c. 6700 km^2^), and the landscape was dominated by agriculture, whereas our study site has a greater diversity of LULC types including significant marine areas and comparably low human populations with low land-use intensity. Large spatial extents are more likely to include more distinct landscape types than smaller spatial extents which in turn will influence ES, ES bundles and the social–ecological system archetypes contained within the landscape (Saidi and Spray 2018; Meacham et al. 2022). Our results indicate that scale has a small effect on ES bundle identification across large spatial scales with clear and strong social–ecological gradients, which is consistent with Madrigal-Martínez and Miralles I García (2020). Our bundles were intuitive in that they followed clear geographical gradients in the region and could be a useful communication tool for stakeholders and institutions (Malmborg et al. 2021). If ES typologies are locally contextualised through engagement with relevant stakeholders concerned with decision making and management as we have done, the ES bundles produced with that typology can be better grasped by those stakeholders (Malmborg et al. 2021). Despite the strong congruence in bundles at the grid and municipal scales, we do emphasise that the overlap is imperfect and identifying the mismatch between underlying social–ecological characteristics at the grid scale and administrative boundaries is important for operationalising our findings for management and planning (Crouzat et al. 2015).

### Ecosystem services across zones

We found differences in relative ES provision between the aggregated transition and core zones, but this difference was not evident between transition and buffer zones. Cultural ES, recreational hunting and fishing in particular, were higher in the aggregated core zone whilst provisioning ES were higher in the transitions and buffer zones (Appendix S2, Fig. S1). Castillo-Eguskitza et al. (2019) also found higher levels of cultural ES supply in core zones than other zones, which in combination with low levels of provisioning ES is consistent with the objective of BRs for biocultural conservation. In contrast, Palliwoda et al. (2021) found that differences in ES supply between transition and buffer zones were more marked although we note that Palliwoda et al. (2021) excluded all marine zones from their analysis. Indeed, when we excluded marine areas from our analysis, we found more variation in the differences in ES supply across zones (Appendix S2, Fig. S2).

In both previous studies, only aggregated zones were considered, yet many BRs comprise multiple individual core and buffer zones, each of which may be dominated by one or few LULCs and the importance of disaggregated zonation assessment has been shown by Cusens et al. (2022), which focussed on the socio-cultural values of ES. Our consideration of multiple ES in individual zones rather than aggregated core and buffer zones identifies important nuances in relative ES supply amongst zones. We highlight that environmental context (social and ecological factors) has a strong influence on relative supply of multiple ES, which is swamped by aggregation, regardless of what type of zone is assessed. Thus, to capture the full breath of biocultural diversity within the BR zones it is crucial to consider zones individually. This argument is similarly identified in recent debates regarding the utility of ‘global maps’ for conservation priority setting (Wyborn and Evans 2021; Chaplin-Kramer et al. 2022).

### Bundles to guide Biosphere Reserve planning

In our study each of the ES bundles contained all or part of at least one core and one buffer zone in addition to transition area, aside from the Bundle 2 at the municipality scale which did not contain any core area. Moreover, the relative ES values we found in our bundles share similarities to those of the ES values in the BR zones and the similarities were at least partially explained by the shared proportions of different LULC in the zones and related bundles (see Appendix S3 Figs. S4 and S5). Despite the relative simplicity of LULC as an indicator, LULC is an important determinant of ES supply and has been shown to be important in explaining the distribution of ES bundles (Meacham et al. 2016). We believe that ES bundles that identify SES archetypes have the potential to guide the planning of BR zonation. The focus on biocultural diversity conservation in BRs means that zonation should focus on the relationships between people and nature, which can be succinctly captured through ES bundles (Meacham et al. 2022). Since ES bundles can in effect capture SES archetypes (Hamann et al. 2015), selecting areas for core and buffer zonation that are representative of the different SES archetypes can contribute to conservation of biocultural diversity. Our assessment of the zonation in NBR fits relatively well with the SES archetypes identified in the bundle analysis with each SES archetype captured in at least one core and one buffer zone. This suggests that based on the different ES and methods we have used for mapping those ES, the zonation has the potential to provide conservation of the biocultural diversity within NBR. However, for this conservation to be realised, there is a need for integrated management across municipalities and scales. Our integrated approach of biophysical and social–cultural methods for assessing ES bundles aligns well with the biocultural diversity focus of BRs and we believe this provides better guidance for addressing the challenges of biocultural conservation goals.

Several authors have already highlighted the potential utility of UNESCO BR organisations to connect diverse stakeholders across spatial and administrative scales (e.g. Olsson et al. 2007; Schultz et al. 2018; Barraclough et al. 2021). This has important implications for ES management and governance due to the cross-scale nature of ES governance, production, management, and use. Management actions and production of ES are often realised at site and/or local scales, whereas regulations governing ES are more common at regional and national levels (Gómez-Baggethun et al. 2013; Raudsepp-Hearne and Peterson 2016). Our multi-scale assessment of ES bundles was important to test for variance of the emergent ES supply levels at different spatial scales at which they are produced, managed, and governed. By identifying that ES supply bundles are relatively stable and similar at grid and municipal scales suggests that actions that affect ES at small spatial scales may emerge and be detectable to a certain degree at larger scales. This can be particularly relevant in NBR because legislature governing land use, and planning and building in Norway are applied nationally but the administration of these acts is decentralised to municipalities (Landbruks- og matdepartementet 1995; Kommunal- og distriktsdepartementet 2008). Recent work on the social network in connection with various activities related to ES has shown that the BR organisation is well connected across many stakeholder groups in NBR, including regional and local government, farmers, hunters and fishers, and industry (Barraclough et al. 2022). This high level of connectivity of the BR organisation combined with our ES bundles has potential to contribute to ES governance within NBR. First, the high level of connectivity can assist in bringing stakeholders involved in natural resources together since BR organisations can act as a bridging institution. Second, the ES bundles can provide an interesting and engaging starting point for stakeholders to contribute to discussions and implementation of co-management of ES across different scales (Malmborg et al. 2021). Third, high connectivity can improve the flow of information between relevant stakeholders and contribute to adaptive governance approaches that is particularly well suited to SES governance and has been successfully implemented in BRs (Olsson et al. 2004, 2007). This is key since highly connected bridging organisations can be particularly effective in networks at identifying wider threats as well as the opportunities to address those threats (Olsson et al. 2007).

### Reflection on our methods

We have considered the proportion of different LULCs within each bundle as a potential explanation for their distribution. Amongst the methods for modelling and mapping ES we have used, many are based on LULC, topographic and other social–ecological characteristics of the landscape (e.g. distance to infrastructure). Any attempts to statistically explain the distribution of the bundles would invariably have used the same variables, or variables derived from those used in the ES mapping. We believe there is a high risk of circularity in reasoning if we had used the same data for predicting the ES as we had used in mapping them (Spake et al. 2017; Saidi and Spray 2018). Further, it is likely that we would increase the error by introducing additional uncertainty on top of the ES models (e.g. Puy et al. 2021). We therefore argue that the explanations with LULC captures a broad range of social–ecological characteristics in the landscape due to the way that strong environmental gradients have shaped the social–ecological landscape and associated land use over millennia.

We have combined an ES mapping and assessment exercise across marine and terrestrial systems. Amongst the ES indicators we have used, many are expressly terrestrial based. This is important to consider since marine resources make important contributions to the economic and cultural character of our study region. Our results should be interpreted with caution in relation to definitive policy or planning decisions related to ES management, particularly in the marine environment. However, we are confident that the patterns we found amongst zones, and the presence and distribution of the bundles would remain or be only marginally different if additional marine-specific ES—aquaculture and commercial fishing most prominently—were included, due the palpable differences in the types of ES supplied by marine and terrestrial systems. Our inclusion of the social-culturally based cultural ES provides an important component for the marine environment. For example, we found that recreational fishing is prominent in the coastal and fjord systems and largely absent from the open ocean in the marine transition zone.

## Conclusion

We integrated biophysical and social-cultural methods for mapping and assessing ES in a multifunctional landscape unified by a UNESCO Biosphere Reserve (BR). The integrated mapping enabled us to undertake a comparative analysis across the zones of the BR and ES bundle assessment that accounted for biocultural diversity, consistent with the objectives of BRs. The analysis of relative ES values amongst zones showed the importance of considering the social–ecological context of the zones and not only their identity (i.e. core, buffer, or transition). We found that the ES bundles were informative in identifying SES archetypes that can inform initial planning of where zones can be established, and guidance for their management in the future. The analysis was undertaken across spatial scales including grid and municipality levels for bundling and, aggregated and disaggregated zones, which is informative for ES co-production, management, and governance since the activities are not constrained to single scales. The value of such research has important implications for BRs since organisations involved in their administration can act as bridges between academia and society, and amongst the actors involved in ES co-production, management, and governance.

## Supplementary Information

Below is the link to the electronic supplementary material.

Supplementary file1 (PDF 1108 kb)
