# Supplementary material for: Integration matters: Combining socio-cultural and biophysical methods for mapping ecosystem service bundles
Source: Ambio. 2023 Feb 13;52(6):1004–21. doi: 10.1007/s13280-023-01830-7 (PMC10160285; doi:10.1007/s13280-023-01830-7)
Supplement: Supplementary file 1 — Supplementary file1 (PDF 1108 kb) [file 13280_2023_1830_MOESM1_ESM.pdf]

Ambio

Electronic Supplementary Material
This supplementary material has not been peer reviewed.

Title: Integration matters: Combining socio-cultural and biophysical methods for mapping ecosystem service bundles

Authors: Jarrod Cusens, Alicia D. Barraclough, Inger Elisabeth Måren

Appendix S1: Methods for mapping ecosystem services

Cultural ecosystem services

We used an approach similar to that of Social Value for Ecosystem Services tool (SolVES; Sherrouse & Semmens, 2020). First, we calculated kernel density surfaces for each cultural ES, then normalised each of these by the maximum cell value among all kernel density surfaces. Second, we extracted the resulting maximum value for each kernel density surfaces. Third, we used Maximum Entropy (MaxEnt) to model probability distributions of each ES using 10 social-ecological landscape characteristics based on previous studies at a resolution of 250 m (Table S 1; Bagstad, Semmens, Ancona, & Sherrouse, 2017; Muñoz, Hausner, Runge, Brown, & Daigle, 2020; Sherrouse, Semmens, & Clement, 2014). We did not remove collinear variables because collinearity does not significantly affect MaxEnt model performance (Feng, Park, Liang, Pandey, & Papeş, 2019). For each MaxEnt probability output, we used the 'minimum training presence threshold' value below which we considered the ES value to be zero (i.e., no capacity to provide the respective ES). Finally, the probability distributions from the MaxEnt models for each ES were multiplied by the maximum kernel density values calculated in step one. All calculations were performed in *R* (R Core Team, 2021) using the *spatialEco* (Evans, 2020) package for kernel density estimates and the *dismo* package (Hijmans, Phillips, Leathwick, & Elith, 2020) for MaxEnt modelling. We used the *ENMeval* package (Kass et al., 2021; Muscarella et al., 2014) for model evaluation and model selection based on Area Under the Curve (AUC) and Akaike Information Criterion corrected for small sample sizes (AICc) respectively. All models performed moderately well (AUC > 0.78).

Table S 1. Data sources used in MaxEnt modelling of cultural ecosystem services from Public Participation GIS data.

Data	Description	Available from
LULC richness	Calculated from union of AR5 and AR50 areal resources layers	AR5 (restricted access) and AR50 (open access) from https://www.nibio.no/
Agricultural land	Calculated from union of AR5 and AR50 areal resources layers	AR5 (restricted access) and AR50 (open access) from https://www.nibio.no/
Forest	Calculated from union of AR5 and AR50 areal resources layers	AR5 (restricted access) and AR50 (open access) from https://www.nibio.no/
Open land	Calculated from union of AR5 and AR50 areal resources layers	AR5 (restricted access) and AR50 (open access) from https://www.nibio.no/
Water (including freshwater and ocean)	Calculated from union of AR5 and AR50 areal resources layers	AR5 (restricted access) and AR50 (open access) from https://www.nibio.no/
Hiking trails	Open street maps	https://www.openstreetmap.org/
Roads	N50 data layer	https://www.geonorge.no/
Buildings	N50 data layer	https://www.geonorge.no/
Elevation	Digital elevation model (DEM) at 10 m resolution	https://www.geonorge.no/
Slope	Calculated from the DEM at 10 m using the 'slope' function in the raster package in R (Hijmans, 2020)	https://www.geonorge.no/

Regulating and maintenance ecosystems services

Habitat quality

We mapped habitat quality separately for terrestrial and marine environments. To map habitat quality in terrestrial environments, we used an approach adapted from Ruas et al. (2021) that accounts for the capacity of different LULC types to support biodiversity, and additionally considered distance from anthropogenic infrastructure and landscape metrics including patch size and contiguity index. For marine environments we used a single value for the capacity of the habitat to support biodiversity and ecological condition potential from The Norwegian Water Resources and Energy Directorate, and additionally considered distance from anthropogenic infrastructure and density of shipping traffic. All landscape metrics were calculated using *landscapemetrics* (Hesselbarth, Sciaini, With, Wiegand, & Nowosad, 2019).

Water retention

Water retention was mapped using the Water Retention Index (Maes et al., 2015; Vandecasteele et al., 2018). This index is a composite indicator of that represents potential of the landscape to retain water and thus regulate potential flooding. The factors included in the indicator comprise retention in vegetation approximated by Leaf Area Index (Copernicus Global Land Services, 2019), retention in soil approximated by soil organic carbon content (Hengl et al., 2017), and retention in groundwater estimated from soil permeability (Panagos, Meusburger, Ballabio, Borrelli, & Alewell, 2014) and bedrock lithology (Gleeson et al., 2011). In addition, slope and soil sealing (Copernicus Global Land Services, 2018) are included since they both influence the capacity of water to be retained and to permeate the ground, respectively.

Avalanche prevention

We mapped avalanche protection capacity of forests using forest structure variables and topographic characteristics from Cordonnier, Berger, Elkin, Lamas, and Martinez (2014). First, we identified avalanche release zones as areas with slopes between 35° and 55°, similarly to Schröter, Barton, Remme, and Hein (2014), and identified areas in release zones that support forest. Then we calculated the Avalanche Protection Index of these avalanche release zones which considers slope and forest characteristics that contribute to reducing avalanche velocity including diameter at breast height, basal area, and dominant tree functional group as either evergreen (*Picea abies*, *P. sitchensis* or *Pinus sylvestris*) or deciduous (*Betula* spp.).

Global climate regulation

We mapped global climate regulation as total ecosystem carbon storage (ton/ha) including above- and below-ground biomass carbon, and soil carbon. Four spatial data layers were used for carbon stock estimation including AR5 and AR50 areal resources (Ahlstrøm, Bjørkelo, & Fadnes, 2019; Flo Heggem, Mathisen, & Frydenlund, 2019) for biomass carbon in non-forested systems, SR16 forest

resources data (Astrup et al., 2019) for biomass carbon in forested systems, and SoilGrids250m (Hengl et al., 2017) for soil carbon. The spatial data from the AR5 and AR50 does not contain carbon estimates so we linked biomass carbon data to the LULC types from several sources (Bartlett, Rusch, Kyrkjeeide, Sandvik, & Nordén, 2020; de Wit, Austnes, Hylen, & Dalsgaard, 2015; Grønlund, Bjørkelo, Hylen, & Tomter, 2010; Grønlund et al., 2008).

Soil retention capacity

The capacity of vegetation to retain soil was modelled and mapped in a similar way to Quintas-Soriano et al. (2019) based on the Revised Universal Soil Loss Equation (RUSLE; Renard, Foster, Weesies, & Porter, 1991) which estimates the amount of soil lost or eroded from land. Inputs for the equation were rainfall erosivity (R; MJ/ha/mm/yr) calculated from mean annual rainfall (Foster, McCool, Renard, & Moldenhauer, 1981), slope length (LS; m) calculated from a digital elevation model, soil erodibility (K; ton/MJ/yr) calculated from SoilGrids250m data (Hengl et al., 2017) and a cropping factor (C; dimensionless) for each LULC type. Then, the capacity of vegetation to retain soil was estimated by calculating the difference between the result of the former from a hypothetical scenario with all cropping factor values set to one (i.e., no soil retention capacity).

Provisioning ecosystem services

Timber and firewood provision

We mapped timber and firewood provisioning capacity as the annual timber increment (m^3/ha/yr) of all forested areas within NBR. We used the species (pine, spruce or birch) and species specific site quality index from SR16 forest resources data (Astrup et al., 2019) to estimate timber increment based on the values from Tveite and Braastad (1981). The site quality index in the SR16 dataset is at a higher resolution (i.e., more site quality classes) than in the one of Tveite and Braastad (1981) so we used a simple linear model to interpolate annual timber increments to the SR16 data.

Water provision

The provision of freshwater was mapped using the Water Yield module in the *Integrated Valuation of Ecosystem Services and Tradeoffs* (InVEST) software (Sharp et al., 2020). The model calculates water runoff with a water balance equation using climatic variables of precipitation and evapotranspiration, soil variables of root restricting layer depth and plant available water content, and average rooting depth of LULC types present in the study area (Table S 4). Additional parameters included are the plant evaporation coefficient (*Kc*) and Z parameter which refers to the seasonal distribution of rainfall. We note that Sharp et al. (2020) advise that water yield data is best interpreted at the watershed or sub-watershed scale rather than the pixel scale. We acknowledge this as a potential issue, but we retain the pixel levels data for consistency with other ES indicators we have mapped.

Cultivated fodder production

We calculated the production of hay (ton/ha) from agricultural statistics and LULC data. First, we used national statistics to estimate the production of hay per hectare in the county in which NBR is located. Then we downscaled this data to grid cells based on the area of agricultural land used for hay production per gird cell, which includes fully- and surface-cultivated soils that can be harvested mechanically. Almost all areas with cultivated soils in NBR (over 99 %) are used for hay and grass production with very little cultivated land for other crops (Statistics Norway, 2019).

Table S2. Data sources used in this study for biophysical modelling and mapping of provisioning, and regulating and maintenance ecosystem services (ES).

ES category	ES	Method	Data source
Regulating and maintenance	Habitat quality	Phenomenological model	Forest data from SR16 forest resources data (Astrup et al., 2019).<br>Non-forest LULC types from union of AR5 (Ahlstrøm et al., 2019) and AR50 (Flo Heggem et al., 2019) areal resources layers.<br>Human infrastructure from N50 database (Kartverket, 2017).<br>Marine ecological condition from The Norwegian Water Resources and Energy Directorate (NVE, 2015)
	Sediment retention	Revised Universal Soil Loss Equation	Rainfall erosivity (R factor) calculated from CHELSA annual rainfall (Karger et al., 2017a, 2017b)<br>Slope length (LS factor) calculated from DEM using RSAGA package (Brenning, Bangs, & Becker, 2018)<br>Soil erodibility (K factor) calculated using data from SoilGrids250m (Hengl et al., 2017)<br>Cropping (C factor) estimated for each LULC from various sources
	Water flow regulation	Water Retention Index	Percent area of water body per catchment (Rwb) calculated from<br>Retention in vegetation (Rv) calculated from Leaf Area Index data from Copernicus Global Land Services (2019).<br>Retention in ground water (Rgw) was calculated from SoilGrids250m (Hengl et al., 2017) based on soil permeability data in Panagos et al. (2014), and bedrock data from the Norwegian Geological Survey and permeability from Gleeson et al. (2011)<br>Slope factor (Rsl) calculated from DEM using RSAGA package (Brenning et al., 2018)<br>Soil sealing (Rss) comes from Copernicus Global Land Services (2018).
	Global climate regulation	Sum of soil carbon and vegetation biomass carbon	Soil carbon data is from SoilGrids250m (Hengl et al., 2017).<br>Forest biomass carbon is from SR16 forest resources data (Astrup et al., 2019).<br>Non-forest LULC types from union of AR5 (Ahlstrøm et al., 2019) and AR50 (Flo Heggem et al., 2019) areal resources layers with carbon data

ES category	ES	Method	Data source
			from various sources (Bartlett et al., 2020; de Wit et al., 2015; Grønlund et al., 2010; Grønlund et al., 2008)
	Avalanche prevention	Avalanche Protection Index	Avalanche release sites were obtained from the Norwegian Water Resources and Energy Directorate (NVE, 2016), slope calculated from the DEM using the slope function in raster (Hijmans, 2020), and forest characteristics were taken from data from SR16 forest resources (Astrup et al., 2019).
Provisioning	Animal fodder production	Downscaled county level data to a grid based on agricultural land cover	Country level fodder production data from the Statistics Norway (2019) and agricultural land cover from AR5 areal resources layer (Ahlstrøm et al., 2019).
	Water supply	InVEST water yield model	Rainfall and evapotranspiration from CHELSA annual rainfall (Karger et al., 2017a, 2017b)<br>Soil rooting depth and plant available water content from SoilGrids250m (Hengl et al., 2017)<br>LULC types from union of AR5 (Ahlstrøm et al., 2019) and AR50 (Flo Heggem et al., 2019) areal resources layers.<br>Watersheds and sub watersheds are from Norwegian Water Resources and Energy Directorate.
	Timber production capacity		Species specific site quality index data from SR16 forest resources (Astrup et al., 2019) and species specific annual tree increment data from Tveite and Braastad (1981).

Appendix S2: Differences in ecosystem service provision among zones

Zones in all habitats

When all habitats are considered together, cultural ES are generally higher in core vs. buffer and buffer vs. transitions zones except for wild plant, berries and mushrooms which was highest in the transition zone and higher in the core vs. buffer zone (Figure S1). Regulating and maintenance, and provisioning ES were generally highest in the buffer zone except for water retention which was highest in the transition zone and habitat quality which was highest in the core and transition zones (Figure S1).

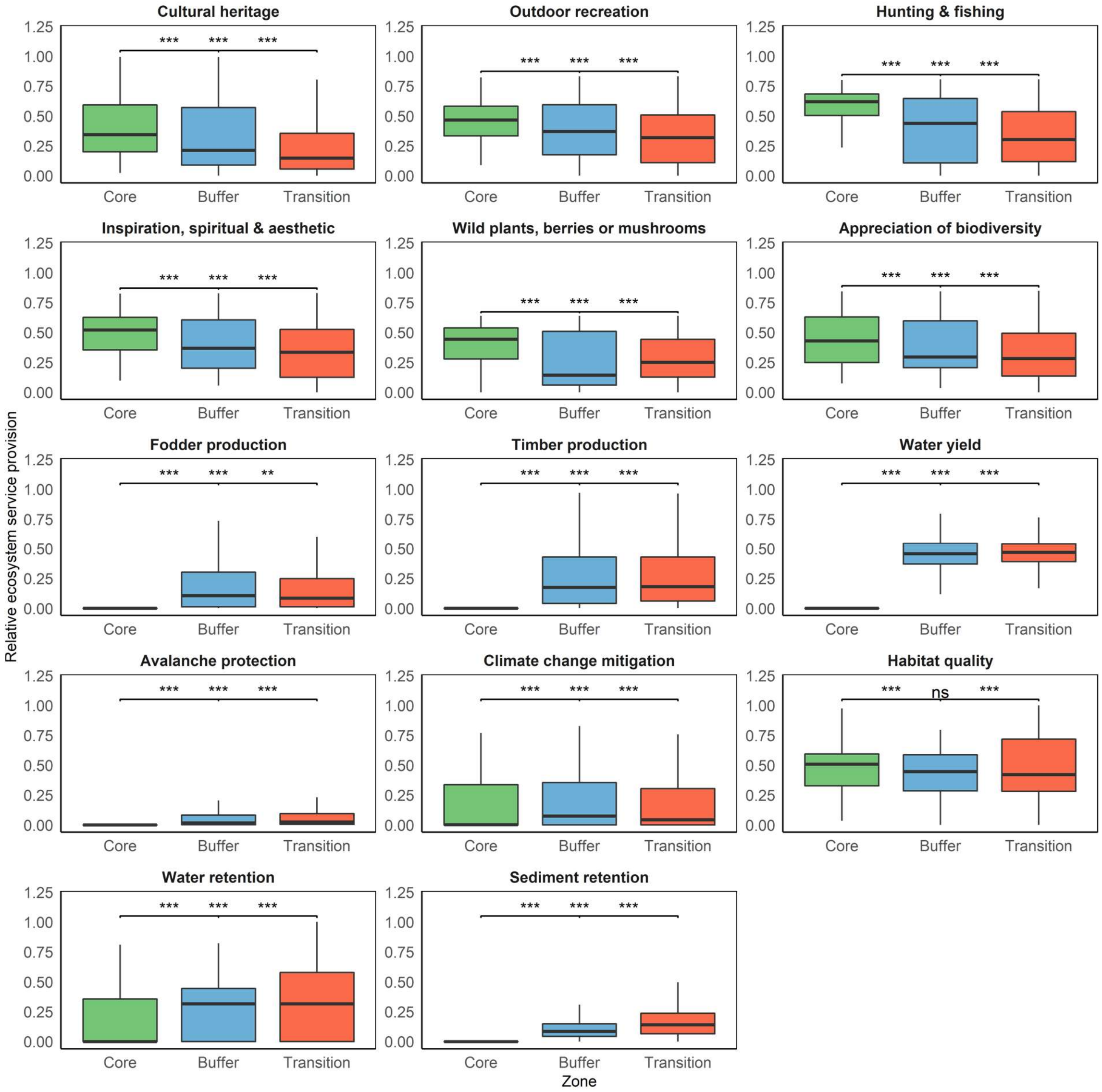

Figure S1. Boxplots of ecosystem service supply among the main zones in all habitats in Nordhordland Biosphere Reserve. Brackets and asterisks represent statistical tests of pairwise comparisons using Wilcox tests. ***, p < 0.0001; **, p < 0.001; ** p < 0.01, ‘, p < 0.05; ns, p > 0.05.

Zones in terrestrial habitats

In terrestrial cultural ES tended to be highest in the transition zone and not different core vs. buffer zone aside from outdoor recreation which was higher in the core vs. buffer zone (Figure S2). Regulating and maintenance, and provisioning ES were more variable.

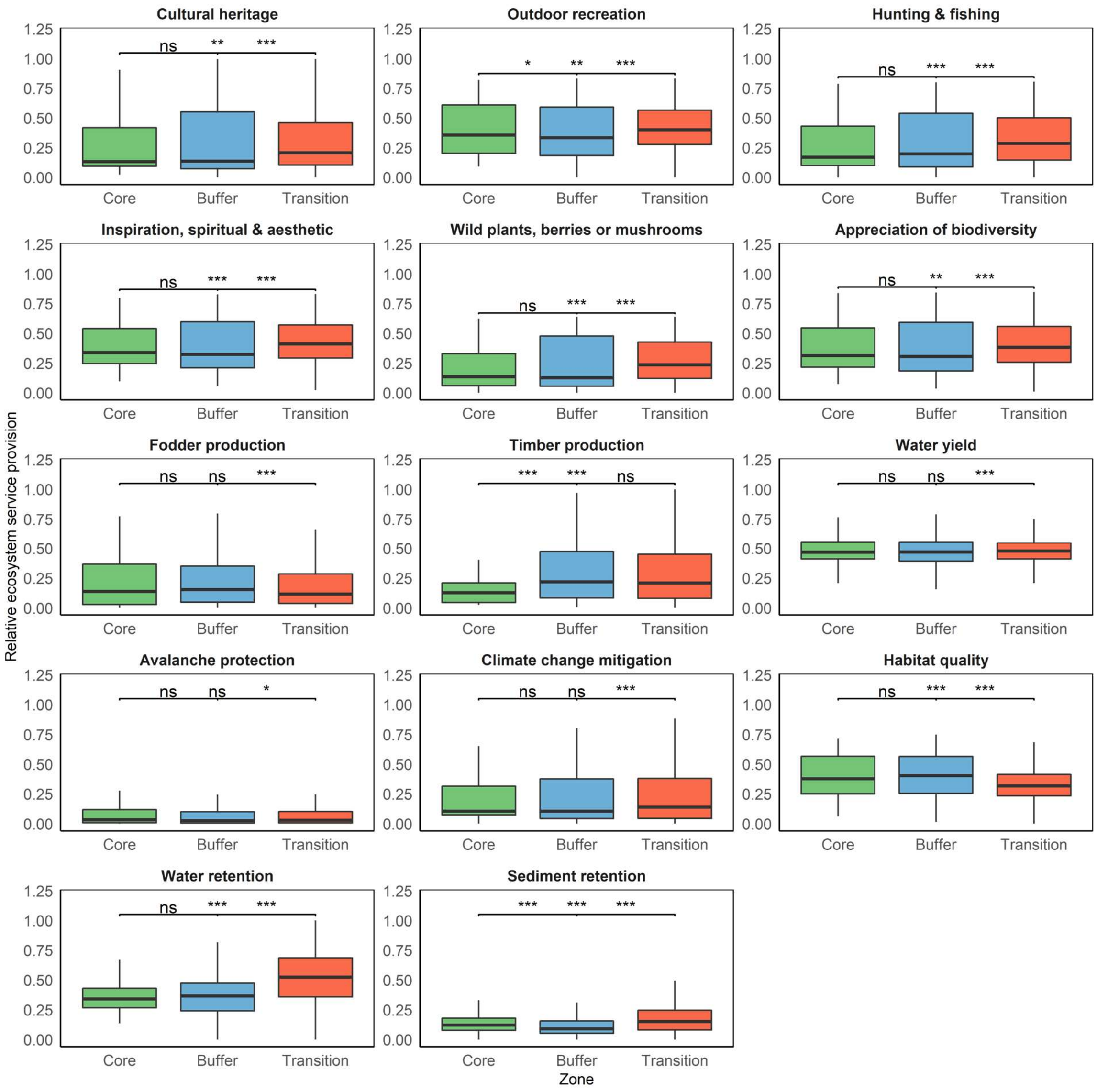

Figure S2. Boxplots of ecosystem service supply among the main zones in terrestrial areas in Nordhordland Biosphere Reserve. Brackets and asterisks represent statistical tests of pairwise comparisons using Wilcox tests. ***, $p < 0.0001$; **, $p < 0.001$; ** $p < 0.01$, ‘, $p < 0.05$; ns, $p > 0.05$.

Zones in marine habitats

In marine habitat cultural ES were highest in the core zone aside from hunting and fishing which was highest in the buffer zone (Figure S3). Likewise, climate change mitigation was highest in the core zone, while Habitat quality was highest in the transition zone (Figure S3)

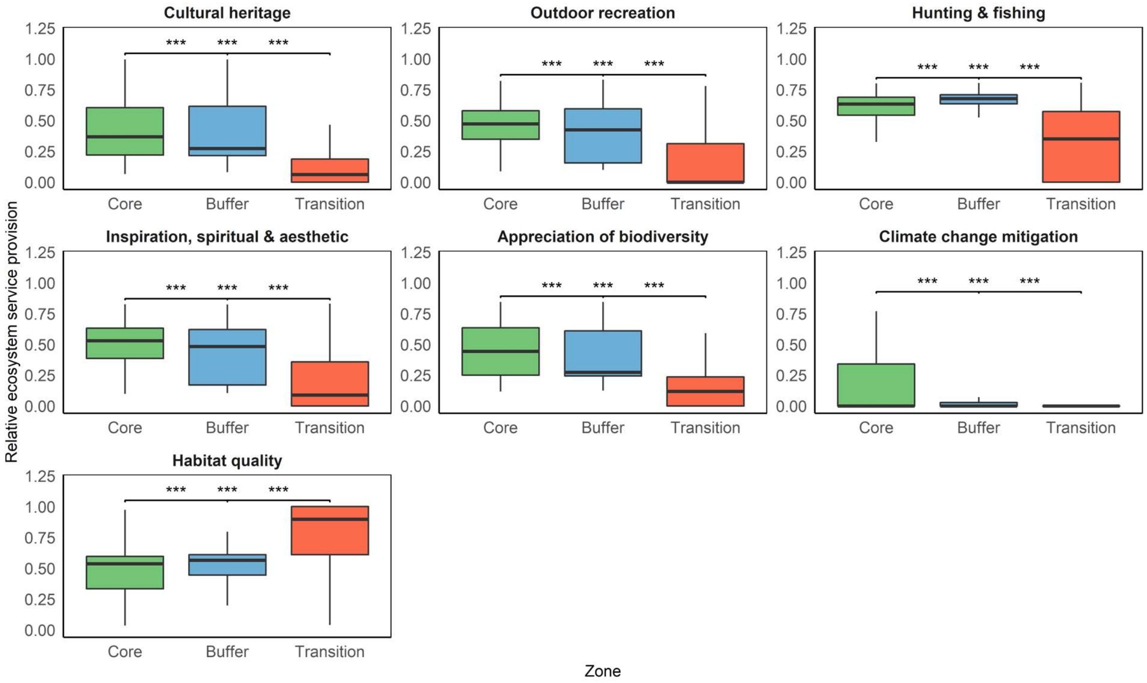

Figure S3. Boxplots of ecosystem service supply among the main zones in marine area of Nordhordland Biosphere Reserve. Brackets and asterisks represent statistical tests of pairwise comparisons using Wilcox tests. ***, p < 0.0001; **, p < 0.001; ** p < 0.01, ‘, p < 0.05; ns, p > 0.05. Note: Fodder production, water yield, timber production, wild plant, berries and mushrooms, sediment retention, water retention and avalanche protection are not shown since these ecosystem services are not provided by marine ecosystems.

Appendix S3: Land Use Land Cover composition of the ecosystem service bundles

The proportions of different LULC in each bundle at both scales were distinctive with Bundle 1 comprising a more even proportion of all LULC types, Bundle 2 being predominantly marine and Bundle 3 being predominantly 'Open and sparse vegetation' and forest (Figure S4). The main differences in LULC between the scales are the complete absence of inland terrestrial areas in Bundle 2 at the municipal scale and the lower proportion of marine areas in Bundles 1 and 3 at the grid scale (Figure S4).

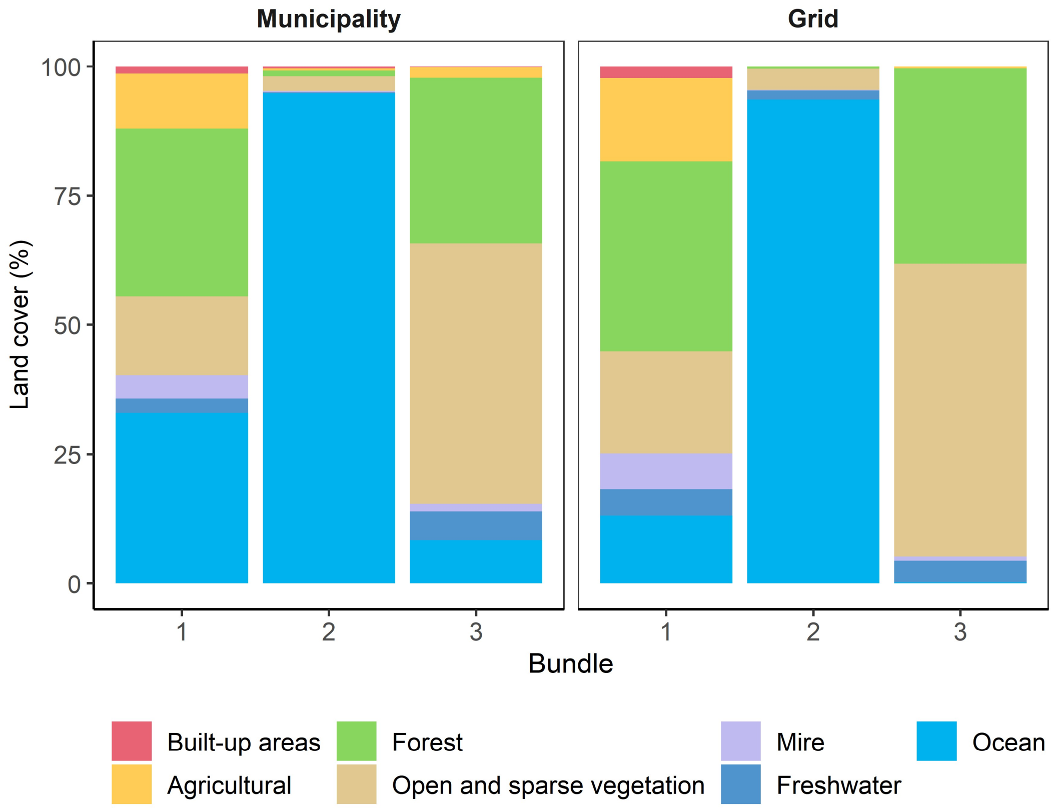

Figure S4. The proportions of the seven main land use-land cover types in each ecosystem service bundle at municipality and grid (250m × 250m) scales.

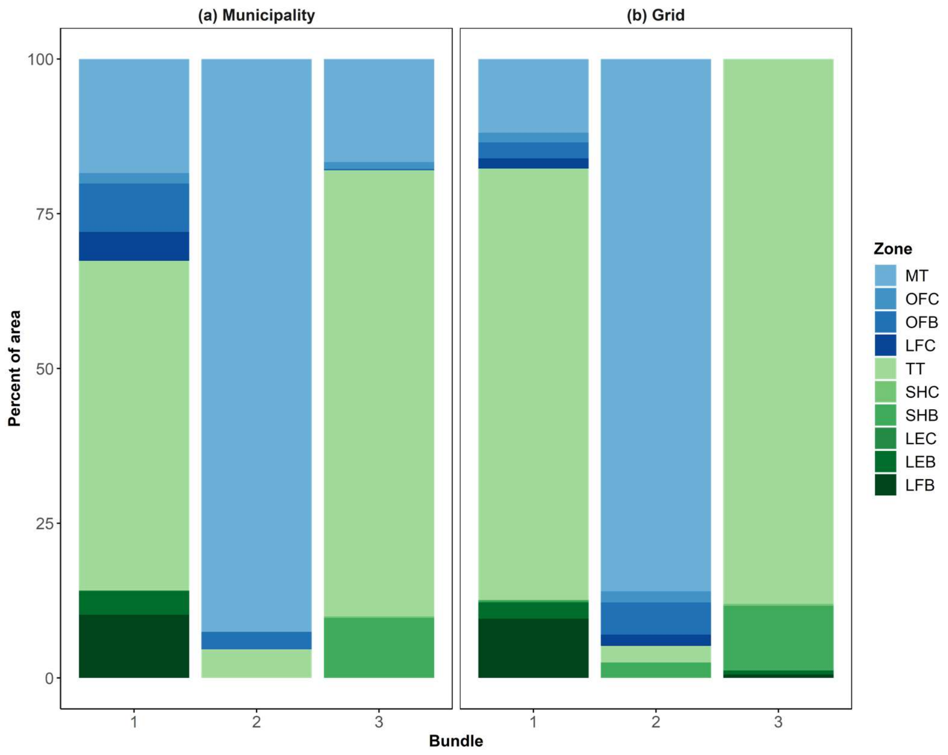

Figure S5. Relative areal proportion of zones within each bundle at the (a) municipality and (b) grid scales. Blue and green represent marine and terrestrial (and one freshwater) zones respectively. MT = Marine transition, OFC = Osterfjorden core, OFB = Osterfjorden buffer, LFC = Lurefjorden core, TT = Terrestrial transition, SHC = Stølsheimen core, SHB = Stølsheimen buffer, LEC = Loneelvi core, LEB = Loneelvi buffer, LFB = Lurefjorden buffer.

References

Ahlstrøm, A. P., Bjørkelo, K., & Fadnes, K. (2019). AR5 classification scheme – classification of areal resources (In Norwegian). *Norsk Institutt for Bioeconomi (NIBIO)*.

Astrup, R., Rahlf, J., Bjørkelo, K., Debella-Gilo, M., Gjertsen, A.-K., & Breidenbach, J. (2019). Forest information at multiple scales: development, evaluation and application of the Norwegian forest resources map SR16. *Scandinavian Journal of Forest Research, 34*(6), 484-496. doi:10.1080/02827581.2019.1588989

Bagstad, K. J., Semmens, D. J., Ancona, Z. H., & Sherrouse, B. C. (2017). Evaluating alternative methods for biophysical and cultural ecosystem services hotspot mapping in natural resource planning. *Landscape Ecology, 32*(1), 77-97. doi:10.1007/s10980-016-0430-6

Bartlett, J., Rusch, G. M., Kyrkjeeide, M. O., Sandvik, H., & Nordén, J. (2020). *Carbon storage in Norwegian ecosystems* (1774). Retrieved from Trondheim, Norway:

Brenning, A., Bangs, D., & Becker, M. (2018). RSAGA: SAGA Geoprocessing and Terrain Analysis. *R package version 1.3.* Retrieved from https://CRAN.R-project.org/package=RSAGA

Copernicus Global Land Services. (2018). High Resolution Layer: Imperviousness Density (IMD) 2018. Retrieved from https://land.copernicus.eu/pan-european/high-resolution-layers/imperviousness/status-maps/imperviousness-density-2018

Copernicus Global Land Services. (2019). *Vegetation Properties: LAI 300m v1*. Retrieved from: https://land.copernicus.eu/global/products/lai

Cordonnier, T., Berger, F., Elkin, C., Lamas, T., & Martinez, M. (2014). Models and linker functions (indicators) for ecosystem services. (ARANGE Deliverable D2.2). Retrieved from http://www.arange-project.eu/wp-content/uploads/ARANGE-D2.2_Models-and-linker-functions.pdf

de Wit, H. A., Austnes, K., Hylen, G., & Dalsgaard, L. (2015). A carbon balance of Norway: terrestrial and aquatic carbon fluxes. *Biogeochemistry, 123*(1), 147-173. doi:10.1007/s10533-014-0060-5

Evans, J. S. (2020). _spatialEco_. *R package version 1.3-1*. Retrieved from https://github.com/jeffreyevans/spatialEco

Feng, X., Park, D. S., Liang, Y., Pandey, R., & Papeş, M. (2019). Collinearity in ecological niche modeling: confusions and challenges. *Ecology and Evolution, 9*(18), 10365-10376. doi:https://doi.org/10.1002/ece3.5555

Flo Heggem, E. S., Mathisen, H., & Frydenlund, J. (2019). AR50 – Arealressurskart i målestokk 1:50 000. Et heldekkende arealressurskart for jord- og skogbruk [AR50 – Area resource map at a scale of 1:50 000. A comprehensive area resource map for soil and forestry]. *Norsk Institutt for Bioeconomi (NIBIO)*.

Foster, G. R., McCool, D. K., Renard, K. G., & Moldenhauer, W. C. (1981). Conversion of the universal soil loss equation to SI metric units. *Journal of Soil and Water Conservation, 36*(6), 355-359.

Gleeson, T., Smith, L., Moosdorf, N., Hartmann, J., Dürr, H. H., Manning, A. H., . . . Jellinek, A. M. (2011). Mapping permeability over the surface of the Earth. *Geophysical Research Letters, 38*(2). doi:https://doi.org/10.1029/2010GL045565

Grønlund, A., Bjørkelo, K., Hylen, G., & Tomter, S. (2010). CO_2-opptak i jord og vegetasjon i Norge: lagring, opptak og utslipp av CO_2 og andre klimagasser [CO_2 uptake in soil and vegetation in Norway: storage, uptake and emissions of CO_2 and other greenhouse gases]. *Bioforsk Rapport, 5*(162/2010).

Grønlund, A., Knoth de Zarruk, K., Rasse, D., Riley, H., Klakegg, O., & Nystuen, I. (2008). Kunnskapsstatus for utslipp og binding av karbon i jordbruksjord [Knowledge status for emissions and sequestration of carbon in agricultural soil]. *Bioforsk Rapport, 3*(132/2008).

Hengl, T., Mendes de Jesus, J., Heuvelink, G. B. M., Ruiperez Gonzalez, M., Kilibarda, M., Blagotić, A., . . . Kempen, B. (2017). SoilGrids250m: Global gridded soil information based on machine learning. *PLOS ONE, 12*(2), e0169748. doi:10.1371/journal.pone.0169748

Hesselbarth, M. H. K., Sciaini, M., With, K. A., Wiegand, K., & Nowosad, J. (2019). landscapemetrics: an open-source R tool to calculate landscape metrics. *Ecography, 42*(10), 1648-1657. doi:https://doi.org/10.1111/ecog.04617

Hijmans, R. J. (2020). raster: Geographic Data Analysis and Modeling. R package version 3.4-5. Retrieved from https://CRAN.R-project.org/package=raster

Hijmans, R. J., Phillips, S., Leathwick, J., & Elith, J. (2020). Dismo: Species Distribution Modeling. R Package Version 1.3.3. Retrieved from https://github.com/rspatial/dismo

Karger, D. N., Conrad, O., Böhner, J., Kawohl, T., Kreft, H., Soria-Auza, R. W., . . . Kessler, M. (2017a). Climatologies at high resolution for the earth's land surface areas. *Scientific Data, 4*(1), 170122. doi:10.1038/sdata.2017.122

Karger, D. N., Conrad, O., Böhner, J., Kawohl, T., Kreft, H., Soria-Auza, R. W., . . . Kessler, M. (2017b). Data from: Climatologies at high resolution for the earth's land surface areas. *Dryad Digital Repository*. doi:https://doi.org/10.5061/dryad.kd1d4

Kartverket. (2017). N50 Kartdata [N50 map data]. Retrieved from https://www.geonorge.no/

Kass, J. M., Muscarella, R., Galante, P. J., Bohl, C. L., Pinilla-Buitrago, G. E., Boria, R. A., . . . Anderson, R. P. (2021). ENMeval 2.0: Redesigned for customizable and reproducible modeling of species' niches and distributions. *Methods in Ecology and Evolution, 12*(9), 1602-1608. doi:https://doi.org/10.1111/2041-210X.13628

Maes, J., Fabrega, N., Zulian, G., Barbosa, A., Vizcaino, P., Ivits, E., . . . C, L. (2015). *Mapping and Assessment of Ecosystems and their Services: Trends in ecosystems and ecosystem services in the European Union between 2000 and 2010*. Retrieved from

Muñoz, L., Hausner, V. H., Runge, C., Brown, G., & Daigle, R. (2020). Using crowdsourced spatial data from Flickr vs. PPGIS for understanding nature's contribution to people in Southern Norway. *People and Nature, 2*(2), 437-449. doi:https://doi.org/10.1002/pan3.10083

Muscarella, R., Galante, P. J., Soley-Guardia, M., Boria, R. A., Kass, J. M., Uriarte, M., & Anderson, R. P. (2014). ENMeval: An R package for conducting spatially independent evaluations and estimating optimal model complexity for Maxent ecological niche models. *Methods in Ecology and Evolution, 5*(11), 1198-1205. doi:https://doi.org/10.1111/2041-210X.12261

NVE. (2015). Vannforekomst - Kyst [Water body - Coast]. Retrieved from https://nedlasting.nve.no/gis/

NVE. (2016). Aktsomhetsområder for skred og flom [Areas of caution for landslides and floods]. Retrieved from https://nedlasting.nve.no/gis/

Panagos, P., Meusburger, K., Ballabio, C., Borrelli, P., & Alewell, C. (2014). Soil erodibility in Europe: A high-resolution dataset based on LUCAS. *Science of The Total Environment, 479-480*, 189-200. doi:https://doi.org/10.1016/j.scitotenv.2014.02.010

Quintas-Soriano, C., García-Llorente, M., Norström, A. V., Meacham, M., Peterson, G. D., & Castro, A. J. (2019). Integrating supply and demand in ecosystem service bundles characterization across Mediterranean transformed landscapes. *Landscape Ecology, 34*(7), 1619-1633. doi:10.1007/s10980-019-00826-7

R Core Team. (2021). R: A language and environment for statistical computing. *R version 4.1.1*. Retrieved from https://www.R-project.org/

Renard, K. G., Foster, G. R., Weesies, G. A., & Porter, J. P. (1991). RUSLE: Revised universal soil loss equation. *Journal of Soil and Water Conservation, 46*(1), 30-33. Retrieved from https://www.jswconline.org/content/jswc/46/1/30.full.pdf

Ruas, S., Ó hUallacháin, D., Gormally, M. J., Stout, J. C., Ryan, M., White, B., . . . James Moran, J. (2021). *Spatial distribution of ecosystem services in Irish landscapes: From habitat quality to food production - analysing current trade-offs and hotspots of ecosystem services in agricultural landscapes*. Retrieved from

Schröter, M., Barton, D. N., Remme, R. P., & Hein, L. (2014). Accounting for capacity and flow of ecosystem services: A conceptual model and a case study for Telemark, Norway. *Ecological Indicators, 36*, 539-551. doi:https://doi.org/10.1016/j.ecolind.2013.09.018

Sharp, R., Douglass, J., Wolny, S., Arkema, K., Bernhardt, J., Bierbower, W., . . . Wyatt, K. (2020). *InVEST 3.9.0 User's Guide.* Retrieved from https://naturalcapitalproject.stanford.edu/software/invest

Sherrouse, B. C., & Semmens, D. J. (2020). *Social Values for Ecosystem Services, version 4.0 (SolVES 4.0)—Documentation and user manual* (7-C25). Retrieved from Reston, VA: http://pubs.er.usgs.gov/publication/tm7C25

Sherrouse, B. C., Semmens, D. J., & Clement, J. M. (2014). An application of Social Values for Ecosystem Services (SolVES) to three national forests in Colorado and Wyoming. *Ecological Indicators, 36*, 68-79. doi:https://doi.org/10.1016/j.ecolind.2013.07.008

Statistics Norway. (2019). Holdings cultivating, by region, contents, year and crop. Retrieved from https://www.ssb.no/en/statbank/table/08646/. Retrieved 18 March 2021, from Statistik sentralbyrå https://www.ssb.no/en/statbank/table/08646/

Tveite, A. B., & Braastad, H. (1981). Site index appraisement for spruce, pine and birch (In Norwegian). *Norsk Skogbruk, 27*, 17-22.

Vandecasteele, I., Marí i Rivero, I., Baranzelli, C., Becker, W., Dreoni, I., Lavalle, C., & Batelaan, O. (2018). The Water Retention Index: Using land use planning to manage water resources in Europe. *Sustainable Development, 26*(2), 122-131. doi:10.1002/sd.1723
